# In Vitro Multiplication and Cryopreservation of *Penthorum chinense* Shoot Tips

**DOI:** 10.3390/life12111759

**Published:** 2022-11-01

**Authors:** Rabbi A. K. M. Zilani, Hyoeun Lee, Elena Popova, Haenghoon Kim

**Affiliations:** 1 Department of Agricultural Life Science, Sunchon National University, Suncheon 57922, Korea; 2 Agricultural Training & Management Development Institute, Kaliakoir 1750, Bangladesh; 3 K.A. Timiryazev Institute of Plant Physiology of Russian Academy of Sciences, Moscow 127276, Russia

**Keywords:** alternative plant vitrification solution, ammonium-free medium, cytotoxicity, droplet-vitrification, endangered species, liquid overlay, regrowth medium

## Abstract

This study provides alternative approaches toward ex situ conservation by means of in vitro seed germination and the multiplication of *Penthorum chinense* Pursh using nodal explants. An overlay of a liquid medium on top of a gelled medium significantly increased the growth of shoots and roots, while the presence of activated charcoal or growth regulators (benzyl adenine and α-naphthaleneacetic acid) decreased the growth. Shoot tips of in vitro plantlets were cryopreserved using a droplet-vitrification method. The standard procedure included preculture with 10% sucrose for 31 h and with 17.5% sucrose for 17 h, osmoprotection with loading solution C4-35% (17.5% glycerol + 17.5% sucrose, w/v) for 20 min, cryoprotection with alternative plant vitrification solution (PVS) A3-70% (29.2% glycerol + 11.7% DMSO + 11.7% EG + 17.4% sucrose, w/v) at 0 °C for 30 min, cooling the samples in liquid nitrogen using aluminum foil strips and rewarming by plunging into pre-heated (40 °C) unloading solution (35% sucrose) for 40 min. A three-step regrowth procedure starting with ammonium-free medium followed by ammonium-containing medium with and without growth regulators was essential for the regeneration of cryopreserved shoot tips. The species was found to be very sensitive to the chemical cytotoxicity of permeating cryoprotectants during cryoprotection and to ammonium-induced oxidant stress during initial regrowth steps. Improvement of donor plant vigor by using apical sections and liquid overlay on top of the solid medium for propagation, improved shoot tip tolerance to osmotic stress and increased post-cryopreservation regeneration up to 64% were observed following PVS B5-85% (42.5% glycerol + 42.5% sucrose) treatment for 60 min. The systematic approach used in this study enables fast optimization of the in vitro growth and cryopreservation procedure for a new stress-sensitive wild plant species.

## 1. Introduction

*Penthorum chinense* Pursh is a perennial herb that occurs in swamps and propagates by means of underground rhizomes and, to minor extent, through seeds [1]. The species belongs to Penthoraceae family, and the genus *Penthorum* has only two accepted species [2]. The plant has potential as a traditional herbal medicine with antioxidant properties and anti-cancer, anti-blebbing and hepatoprotective actions, as well as a food supplement [3,4,5,6].

Decreasing populations, habitat loss and climate change are major threats to this species [7]. Moreover, its high medicinal and ornamental value make this species worthy of integrated conservation interventions [7]. In addition to in situ conservation efforts, in vitro seed germination and in vitro culture of this valuable wild species can be considered as complementary strategies [8]. Pence et al. [9] also emphasized the importance of in vitro propagation methods in providing materials for the reintroduction and restauration of populations, and research in wild species that produce limited amounts of seeds.

Cryopreservation is a long-term method of storing living biological materials in liquid nitrogen (LN, −196 °C), after which the conserved samples may be recovered to produce new plants [10]. Cryopreservation combined with in vitro technologies offers a solid basis for developing effective conservation and restoration strategies for endangered species producing seeds that are non-orthodox, dormant or insufficient in quantity [8,9,11].

In a modern cryopreservation method called droplet-vitrification, samples are subjected to a series of pre-LN treatments with progressively increasing concentrations of cryoprotectants (CPAs). After rewarming (post-LN), this process is repeated “in reverse” (from a higher to lower concentration of CPAs) when samples are bathed in an unloading solution and then transferred to a regrowth medium [12]. To simplify the procedure and avoid laborious condition screening at both the pre-LN and post-LN stages, we developed a systematic approach that covers a range of plant species and starts with a limited number of standard procedures using alternative vitrification solutions (VSs) to adopt the cryopreservation protocol to new taxons [12]. This approach was successfully tested with a number of endangered plants, e.g., *Kalopanax septemlobus* (Thunb.) Koidz. [13], *Betula lenta* [14], *Castilleja levisecta* Greenm. [15], *Aster altaicus* var. *uchiyamae* [16] and *Lupinus rivularis* Douglas ex Lindl. [17] with over 60% of average regrowth achieved for all species.

In order to develop a droplet-vitrification protocol for a given plant material, it is necessary to evaluate the sensitivity of samples to cytotoxicity induced by cryoprotection with highly concentrated VSs, which is the main limiting factor for high post-cryopreservation regrowth [12]. Hence, optimization of cryoprotection conditions is crucial in cryopreservation studies. Therefore, the majority of the cryopreservation studies for the new species are focused on the optimization of preculture and cryoprotectant treatments [10]. Regretfully, very few studies have explored the effect of post-cryopreservation (regrowth) conditions (light, medium composition and growth regulators); however, at least for some species, these factors may have a major impact on the regeneration of healthy plantlets from cryopreserved materials [16,18,19]. Thus, in this study we also investigated the effect of sequential regrowth steps and ammonium-free regrowth medium to improve the regeneration of healthy plantlets from cryopreserved shoot tips.

To the best of our knowledge, this is the first report on developing a complementary conservation approach for *Penthorum chinense* through in vitro seed germination, in vitro propagation, and cryopreservation of shoot tips of in vitro propagated plantlets.

## 2. Materials and Methods

### 2.1. Plant Material

#### 2.1.1. In Vitro Seed Germination and Establishment of In Vitro Culture

A small quantity (about 0.1 g) of mature seeds of *Penthorum chinense* Pursh were received from the National Institute of Biological Resources, Incheon, Republic of Korea. For the in vitro germination, the seeds were sterilized with 1% (*v*/*v*) NaOCl for 7 min and then washed with sterile distilled water for 10 min. Then, the seeds were inoculated on Murashige and Skoog (MS) medium [20] with 30 g L^−1^ sucrose, 3.5 g L^−1^ gelrite and 1 g L^−1^ activated charcoal, pH 5.8 (hereafter referred to as ‘MSF’) in 300 mL Gaooze^TM^ culture vessels (Korea Scientific Technology Industry, Suwon, Korea). Seeds were germinated in a culture room at 25 °C under a 16/8 h light/dark photoperiod and 40 µE m^−2^ s^−1^ light intensity (one fluorescent lamp, 40 W).

The germinated plantlets were cultured for six weeks on the same medium at 25 °C under a 16 h photoperiod and 60 µE m^−2^ s^−1^ light intensity (two lamps) and further propagated using nodal sections. To facilitate the growth of healthy plants, 15 mL of liquid MSF medium was added on top of the solid medium at day 10 of every subculture. Developed plants were further propagated using nodal segments (6/vessel) with 7-week subculture intervals.

#### 2.1.2. In Vitro Propagation Using Nodal Segments

To establish the in vitro propagation system, six combinations of in vitro culture conditions were examined, including MS medium strength (full, 1/2), activated charcoal (AC, 1.0 g L^−1^), overlay of liquid medium on top of the gelled solid medium at day 10 (Liquid), growth regulators (benzyl adenine (BA) 0.7 mg L^−1^ + α-naphthaleneacetic acid (NAA) 1.0 mg L^−1^), light intensity 1 or 2 lamps (40 and 60 µE m^−2^ s^−1^, respectively). The medium was supplemented with 30 g L^−1^ sucrose, and pH of the medium was adjusted to 5.8 prior to autoclaving. Nodal cuttings (5–6 mm in length) were inoculated into Gaooze^TM^ culture vessels (seven cuttings per vessel). Three replicates were used for each treatment and all the experiments were repeated thrice. The height (cm) and dry weight (g) of shoots and roots were individually measured after six weeks. Dry weight was measured after drying plant samples to a constant weight at 7 h at 75 °C in an oven.

### 2.2. Droplet-Vitrification Cryopreservation Procedure

#### 2.2.1. Standard Droplet-Vitrification Procedure

Shoot tips (1.3 mm in length, 1–2 lateral leaves) were extracted from 3–4-day-old node cuttings. Except where otherwise stated, explants were precultured in a liquid MS medium with 10% sucrose (S-10%) and 17.5% sucrose (S-17.5%) for 31 h and 17 h, respectively, osmoprotected with C4-35% solution (17.5% glycerol + 17.5% sucrose) at 25 °C for 20 min, and then cryoprotected with vitrification solution A3-70% (29.2% glycerol + 11.7% dimethyl sulfoxide + 11.7% ethylene glycol + 17.4% sucrose) on ice for 30 min.

Shoot tips were then placed in 5 µL droplets of A3-70% on aluminum foil strips (7 mm × 20 mm), before being plunged directly into LN for a minimum of 1 h. For rewarming and unloading, foil strips containing the shoot tips were transferred to 20 mL pre-heated (40 °C) 35% sucrose (S-35%) solution and kept for 40 min, with sucrose solution been replaced by the new solution after the first 15 min. Cryoprotected control (LNC) shoot tips were treated with the same procedure except without cooling in LN, and unloaded in S-35% solution before being transferred to recovery medium. The explants retrieved from S-35% were blotted dry and transferred to recovery medium 1 (ammonium nitrate (NH_4_NO_3_)-free MS medium + 1 g L^−1^ casein hydrolysate + 1 mg L^−1^ gibberellic acid (GA_3_) + 0.5 mg L^−1^ BA with 30 g L^−1^ sucrose and 3.5 g L^−1^ gelrite, hereafter referred to as “RM1”) in SPL culture vessels (90 mm × 40 mm, SPL Life Sciences, Gyeonggi-do, Korea). Shoot tips were kept at 25 °C in the dark for recovery. After 5 days, explants were moved to recovery medium 2 (normal (NH_4_NO_3_-containing) MS medium + 1 g L^−1^ casein hydrolysate + 1 mg L^−1^ GA_3_ + 0.5 mg L^−1^ BA with 30 g L^−1^ sucrose and 3.5 g L^−1^ gelrite, RM2) and cultured for 3 weeks under the same conditions described above for establishment. Developed shoots were further transferred to growth hormone-free MS medium (MSF) for further regeneration.

#### 2.2.2. Experimental Design in Droplet-Vitrification Procedure

A set of 16 treatments was designed to optimize the droplet-vitrification protocol based on analysis of the sensitivity of the material to various treatments (standard procedure, 11 pre-LN and 4 post-LN variants). The variants tested are listed in Table 1. Other conditions remained the same as in the standard procedure (indicated as “standard” in Table 1). The composition of the cryoprotectant (CPA) solutions is given in Table 2. For the treatment of container modification, shoot tips were cryopreserved in 2 mL cryovials with 0.5 mL VS A3-70%, kept in LN for 1 h and rewarmed in a pre-heated (40 °C) water bath; shoot tips were withdrawn from the vials, unloaded in S-35% solution and recovered as described above in the standard droplet-vitrification procedure.

#### 2.2.3. Further Optimization of Droplet-Vitrification Procedure

To improve the LN regeneration, subcultured plantlets were revitalized by using of apical section, instead of nodal section, and applying of liquid overlay (MSF medium) on top of gelled medium after 2 weeks of inoculation. After two cycles of 5–6 weeks subcultures, shoot tips were extracted from 3–4-day-old node cuttings and subjected to cryopreservation using the standard droplet-vitrification procedure. Based upon the results in first round experiments, both four-component VS of “A” series (PVS2, AS-70%, A3-80%) were cryoprotected on ice, and a PVS3 dilution (B5-85%) was treated at room temperature. The composition of each VS was listed in Table 2.

#### 2.2.4. Assessment of Shoot Tip Recovery and Statistical Analysis

Survival was evaluated two weeks following cryopreservation by counting the number of shoot tips showing elongation, or formation of new leaves. Regeneration (shoot development) was determined after 8 weeks, when the shoot tips had developed into normal plantlets (≥8 mm) with fully developed leaves and roots.

Ten to thirteen shoot tips were used per experimental conditions and the experiments were replicated 3–4 times. Data were analyzed by analysis of variance (ANOVA) and Duncan’s multiple range test (*p* < 0.05) following arcsine transformation using SAS 9.1 software (SAS, Raleigh, NC, USA). Results are presented as average values with their standard deviation.

## 3. Results

### 3.1. Establishment of In Vitro Culture and Propagation System

To establish the in vitro propagation system, nodal segments were cultured in six condition variants (standard + 5 alternative conditions; Table 1) for 6 weeks. Significant differences in growing pattern were found among the treatments after 4–5 weeks. Half-strength MS medium (treatment 1 in Table 3) resulted in similar or slightly higher length of shoots and roots and dry weight of shoots and roots compared to the full-strength MS medium (treatment 2), though the differences were insignificant (Table 3). An overlay of liquid MSF medium on top of gelled medium (treatment 3) produced significantly greater length of shoots and roots compared to gelled medium only, while the dry weight of shoots and roots was not significantly different. Removing of the activated charcoal (treatment 4) had a positive effect on the length of shoots (21.6 vs. 11.2 cm) and roots (11.6 vs. 4.1 cm), while dry weight of shoots were not significantly affected. Addition of growth regulators (treatment 5) showed a notable negative effect on the in vitro growth of shoots and roots. Light intensity (one vs. two lamps) was not a significant factor for in vitro growth (treatment 2 vs. treatment 6).

In conclusion, conditions combining the most favorable factors: half-strength MS medium without AC with an overlay of liquid medium on top of solid medium with two lamps (60 µE m^−2^ s^−1^) was recommended for the in vitro culture of *P. chinense* plantlets.

### 3.2. Droplet-Vitrification Procedure for Shoot Tip Cryopreservation

#### 3.2.1. Effect of Preculture

Among the preculture treatments tested, preculture with moderate sucrose concentrations (S-10% and S-17.5%) did not significantly improve the survival and regeneration of both cryoprotected (LNC) and cryopreserved (LN) shoot tips, compared to treatment without preculture (No-PC) (Table 4). Preculture with higher sucrose concentration (S-25%) produced even lower survival and regeneration of both the cryoprotected control (LNC, 35.1% survival, 18.7% regeneration) and cryopreserved (LN, 29.3% survival, 8.2% regeneration) shoot tips. This study suggests that *P. chinense* shoot tips are sensitive to osmotic stress and, moreover, preculture with moderate concentration of sucrose (S-10 and S-17.5%) was not effective for the acquisition of osmotic adaptation to further cryoprotectant treatments.

#### 3.2.2. Effect of Osmoprotection and Container in Cooling/Warming

Even after the best preculture treatment based on Table 4, shoot tips were sensitive to osmotic stress induced by direct exposure to VS A3-70% without osmoprotection (Table 5, No-OP) with 52.2% survival and 13.3% regeneration before cryopreservation (LNC). After liquid nitrogen exposure (LN), survival and regeneration level were similar to those of cryoprotected control (LNC), indicating that the low shoot tip viability was caused by osmotic stress induced by direct exposure to VS, rather than freezing injury. Application of osmoprotection solution (OP) for 20 min significantly (*p* < 0.05) increased survival and regeneration of LNC shoot tips.

Cooling and rewarming using 2 mL cryovials (Vial) produced 45.2% lower survival and 22.5% lower regeneration compared to the aluminum foil strips (Table 5), which indicated that shoot tips were insufficiently cryoprotected via standard treatment with VS A3-70% before being immerged into LN.

#### 3.2.3. Effect of Cryoprotection Treatment (Vitrification Solution)

When cryoprotection was performed with PVS2 and its variants, four-component vitrification solutions of “A” series, on ice for 30 min, although there was no significance, a relatively lower rate of survival of both LNC and LN shoot tips was observed with original PVS2 (A1-73.7%) and the alternative A3-90%, possibly due to their cytotoxicity (Table 6). Dilution of VS A3-90% to 70% (A3-70%) produced the highest regeneration of both LNC (48.3%) and LN (49.8%) shoot tips. Longer cryoprotection (60 min) with A3-70% also resulted in lowest regeneration of both LNC (31.1%) and LN (26.2%), reflecting the cytotoxicity. Original PVS3 (B1-100%) was also toxic to shoot tips (Table 6), and thus its dilution to 80% (B5-80%) improved the regeneration of LNC and LN shoot tips by 16.6% and 8.4%, respectively.

Although there was no significance, PVS2 and its variants produced slightly higher LN regeneration compared to PVS3 and its variant (36.8–49.8% vs. 32.5–40.9%). Among the VSs tested, the highest survival and regeneration of LNC and LN shoot tips were recorded with a dilution of A3-90% to 70% (A3-70%) for 30 min (Table 6), indicating that *P. chinense* shoot tips are very sensitive to both osmotic stress and chemical toxicity induced by highly concentrated VSs during cryoprotection.

#### 3.2.4. Effect of Step-Wise Regrowth on Different Media

Overall, the regrowth conditions tested moderately affected the survival of both LNC and LN shoot tips, and significantly (*p* < 0.05) affected the regeneration of both LNC and LN treatments. Initial regrowth on ammonium-free medium for 5 days (RM1) followed by standard medium supplemented with growth regulators (RM2) and, finally, hormone-free medium (MSF) (Table 7, RM1-RM2-MSF, standard) showed relatively high regeneration (43.6% LNC and 35% LN). A more frequent transferring to a new medium of RM2 or MSF (RM1-RM2-RM2-MSF, RM1-RM2-MSF-MSF) did not significantly improve the regeneration. Initial regrowth on ammonium-containing medium with growth regulators without transferring to a new medium (RM2~) was harmful for the regeneration of both LNC and LN (0.0% and 3.3%, respectively). Similarly, initial regrowth with ammonium-containing medium with growth regulators followed by the same transfer steps as in the standard treatment (RM2-RM2-MSF) produced poor regeneration of LNC 10.0% and LN 5.2%. These results suggest that the initial regrowth with ammonium-containing regrowth medium was always harmful for the regeneration of LNC and LN shoot tips. Moreover, sequential transfer to fresh regrowth medium, per se, was not beneficial for improving the regeneration of both LNC and LN shoot tips. Hence, initial (5 days) regrowth on ammonium-free medium followed by step-wise transfer to ammonium containing medium and then medium without growth regulators was beneficial for regeneration of normal plants after cryoprotection and cryopreservation.

### 3.3. Further Optimization of Droplet-Vitrification Procedure

**Figure 1:**
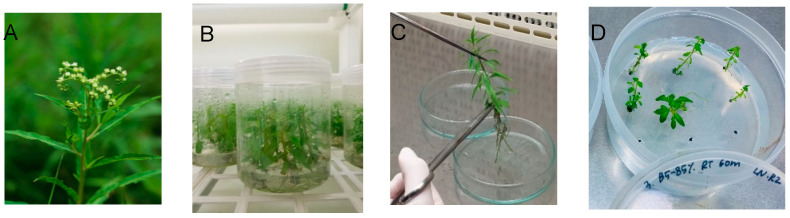
(**A**), *Penthorum chinense* plant in wild habitat; (**B)**, in vitro-cultured plantlets under the subculture condition of MSF for six weeks; (**C**), in vitro-grown plantlet before node cutting; (**D**), regrowth of cryopreserved shoot apices following cryoprotection with B5-85% RT for 60 min and RM1-RM2-MSF regrowth using SPL cultures (90 mm × 40 mm) for six weeks.

**Figure 2:**
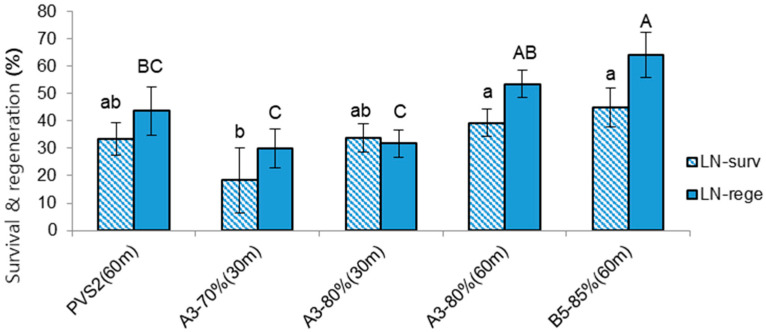
Effect of cryoprotection (vitrification solution composition and conditions of treatment) on survival (surv) and regeneration (rege) of cryopreserved (LN) *Penthorum chinense* shoot tips. See Table 2 for composition of vitrification solutions; m—minutes. Different letters (a,b for LN-surv; A,AB,BC,C for LN-rege) on each graph differ by Least Significant Difference Test (*p* < 0.05).

As an attempt to increase regeneration after cryopreservation, we modified the subculture of donor plants by inoculating the apical section, instead of the nodal section, and applying liquid overlay on top of the gelled medium. Donor plants subcultured in this manner were vitalized, and their subculture duration could be reduced from 7 to 5–6 weeks (Figure 1B,C). Shoot tips excised from these revitalized node cuttings were more tolerate to osmotic stress and chemical toxicity of CPAs and thus the duration of osmoprotection treatment with C4-35% could be increased from 20 min to 40 min. At the same time, the concentration of the VS and cryoprotection duration were also increased. With these modified conditions, the highest regeneration after cryopreservation (64.2%) was produced using cryoprotection with VS B5-85% for 60 min. The second-best treatment (53.6% regeneration) applied VS A3-80% for 60 min (Figure 2). The regeneration of shoot tips cryopreserved after cryoprotection with A3-70% for 30 min (30%) or A3-80% for 30 min (31.9%) was slightly lower than the values for the previous experiments, i.e., 49.8% or 36.8%%, respectively. This result reflects that vigorously grown donor plants allowed a higher concentration and longer duration of osmoprotection and cryoprotection treatments. The cryopreserved shoot tips following the cryoprotection with B5-85% for 60 min were regrown to become normal plantlets with a step-wise regrowth medium of RM1-RM2-MSF (Figure 1D).

## 4. Discussion

### 4.1. In Vitro Culture and Propagation System

The in vitro germination of *P. chinense* seeds and the multiplication of plantlets using node sections as well as cryopreservation were investigated in this study as an alternative ex situ conservation approach for this species.

Among various in vitro culture media and conditions tested, an overlay of liquid medium on top of gelled medium most effectively promoted the growth of shoots and roots, while adding activated charcoal or growth regulators was not beneficial. Pullman and Skryabina [21] reported that an overlay of liquid medium on gelled medium 14 days after subculture improved embryogenic tissue initiation in conifers, possibly due to allowing nutrients replenishment, adjustment of pH, hormones, etc. Liquid overlay also stimulated the multiplication of ginger plantlets [22]. In the combination of solid and liquid media, direct contact with the liquid medium may promote the growth, particularly when the root system and its function are not sufficiently developed. When node cuttings were cultured for the in vitro tuberization of *Solanum tuberosum* (L.) cultivars, a liquid overlay on top of the solid medium produced significantly greater length and dry weight of shoots and roots compared to the other treatments, i.e., solid (gelled), static liquid, and wall-supported liquid media (unpublished data). Gelling agents may affect the physiochemical characteristics of the culture medium due to differences in the diffusion rate of nutrients, elemental and organic impurities and gel hardness [23]. As a gelling agent, gelrite was used in this study since it supported faster growth of shoots compared to agar, perhaps due to impurity of agar which contains agropectin [24]. Agar gelation also slowed the absorption of 2,4-dichlorophenoxyacetic acid (2,4-D) and abscisic acid (ABA) compared to gelrite [21].

Activated charcoal is often used in tissue culture to improve tissue growth and development via, among other factors, the absorption of inhibitory compounds in the culture medium [25]. In several orchid, yam and *Lycium* species, the addition of AC enhanced multiplication by increasing plant height, rooting and protocorm development from seeds [26,27,28], but inhibited plant regeneration in *Athyrium niponicum* var. pictum [29]. Addition of AC in regrowth medium had a beneficial effect on the recovery of cryopreserved *Lavandula* cells [30] and Norway spruce somatic embryos [31]. In the present study, the adding of AC was designed as the standard condition, since AC was beneficial for the formation of normal plantlet during the in vitro germination and in vitro culture establishment stages in preliminary experiment. However, this study revealed that the AC had a negative effect on the length and dry weight of *P. chinense* plantlets after 6-week subculture, possibly through the absorption of needed substances (vitamins, minerals, etc.) or some other, still unknown, reasons. In conclusion, a combination of half-strength MS medium without AC with an overlay of liquid medium with two lamps (60 µE m^−2^ s^−1^) was recommended for the in vitro multiplication of *P. chinense* plantlets.

### 4.2. Development of Droplet-Vitrification Protocol

For the acquisition of osmotic tolerance, preculture treatment to a final concentration of 0.3 M (10% w/v) or 0.5 M (17.5% w/v) sucrose has usually been applied in vitrification procedures [32]. Preculture with high concentration of sucrose affected cell metabolism, causing alterations in gene expression [33], the accumulation of specific amino acids and soluble sugars [34,35,36], and changes in protein and fatty acid composition [33,37].

In this study, the regeneration of cryopreserved *P. chinense* shoot tips was not significantly improved by stepwise preculture with 10% sucrose → 17.5% sucrose. A higher concentration of sucrose in preculture medium (10% sucrose → 25% sucrose) was even detrimental for regrowth (Table 4). This indicates that the species is sensitive to osmotic stress, even imposed by sucrose treatment. Likewise, shoot tips of another endangered wild species habituated in wetland, *Pogostemon yatabeanus*, were very sensitive to osmotic stress, and 10% sucrose was chosen as the best preculture condition [38]. Mallón et al. [39] noted that preculture with 0.25–0.3 M sucrose was effective for regrowth, while preculture with a higher sucrose concentration resulted in reduced regrowth of cryoprotected shoot tips in critically endangered species of the Asteraceae family.

Osmoprotection (or loading) treatment (incubating the explants with moderately concentrated CPAs before vitrification solution treatment) increased the osmotic tolerance of the cells and minimize osmotic damage caused by the VS [40,41,42]. Though a mixture of 2 M glycerol (18.0%) plus 0.4 M sucrose (13.7%) has been most frequently used as an osmoprotectant solution [43], an alternative formulation, C-35%, produced higher LN regeneration in osmotically sensitive materials, such as chrysanthemum shoot tips [44]. Osmoprotection was particularly important for hairy roots of *Rubia akane*, which have been shown to be highly susceptible to the cytotoxicity of VSs [45]. In the present study, osmoprotection treatment with C4-35% for 20 min increased regeneration of cryoprotected (21.7%) and cryopreserved (18.3%) shoot tips (Table 5), but there was no significant difference in regeneration. In the preliminary experiment, a longer loading treatment (40 min) was detrimental. Alternatively, a sequential osmoprotection from lower to higher concentration or longer exposure at 0 °C instead of 25 °C might be beneficial for a sufficient osmoprotection of sensitive material [46].

Cytotoxicity of the highly concentrated VSs is a limiting factor for the successful regeneration of differentiated propagules such as shoot tips; therefore, identifying the nature of cytotoxicity, whether biochemical and/or osmotic [47], is necessary for establishing the reliable solution-based vitrification protocols for a given plant material. Although PVS2 [32,48] is the most commonly used VS, PVS3 is recommended for larger explants [32]. Additionally, their alternative variants were also successfully tested [49]. Dilution of a PVS2 variant, A3-90%, to 80% (A3-80%) or 70% (A3-70%) has been successfully applied for sensitive materials, such as tiny shoot tips of *Pogostemon yatabeanus* (Makino) Press [35], *Aster altaicus* [16], or root cultures of *Rubia akane* [50]. In the present study, *P. chinense* shoots were very sensitive to both osmotic stress and chemical toxicity induced by original PVS3, PVS2 or its variant A3-90%, and thus cryoprotection with the diluted VS B5-80% or A3-70% for 30 min produced higher survival and regeneration of both LNC and LN shoot tips. Based on the classification proposed in our previous research [12], *P. chinense* shoots are very sensitive to osmotic stress and chemical cytotoxicity.

Compared to ‘classical’ vitrification using cryovials, droplet-vitrification using aluminum foil strips resulted in higher survival and regeneration of *P. chinense* shoots. With insufficient cryoprotection using VSs of a lower concentration and a relatively shorter duration, higher cooling and rewarming rates provided by aluminum foil strips and a pre-heated (40 °C) unloading solution (droplet-vitrification) were superior to using cryovials (vitrification) and ensured the inhibition of crystallization and recrystallization events [51].

To obtain the high survival and fast regrowth of cryopreserved shoot tips, congenial regrowth conditions are essential; in particular, the initial recovery media and culture conditions are critical in many species [16,18,30]. Recovery could eventually occur in initial darkness, since dark incubation contributed to the avoidance of photo-oxidation, which could be harmful to the tissue [41]. Though plant growth regulators have been widely applied for the recovery of cryopreserved shoot tips [52,53], sequential transferring to a hormone-free medium was beneficial for the normal regeneration of both cryoprotected and cryopreserved shoot tips of *Aster altaicus* [16]. The viability of cryopreserved rice cells exponentially depended on the concentration of ammonium ions on the liquid regrowth medium during the initial seven days after slow freezing [54]. Substitution of ammonium nitrate (NH_4_NO_3_) by potassium nitrate (KNO_3_) improved post-cryopreservation recovery from 22.5% to 53% in *Betula pendula* shoot tips [30]. In the PVS2-based vitrification method, recovery of LN sweet-potato shoot tips was significantly improved by initial regrowth on ammonium-free regrowth medium (32% vs. 93%) [55].

The present study highlights the critical effect of ammonium-free medium at the initial regrowth stages on the successful recovery of cryoprotected and cryopreserved shoot tips in three-steps regrowth procedure. Initial regrowth on the ammonium-containing medium (treatments RM2-RM2-MSF and RM2~ in Table 7) produced very low regeneration of both cryoprotected (0–10%) and cryopreserved (3.3–5.2%) shoot tips. Though the mechanism of the critical effect of ammonium during the first recovery steps is not yet clear, it is hypothesized that any type of injury during cryopreservation, i.e., osmotic stress, chemical toxicity, crystallization, etc. may be associated with oxidative stress. In this case, the initial regrown on ammonium-containing medium triggers ammonium-induced oxidative stress, which amplifies the stress already caused by cryopreservation and causes a failure in recovery of both the LNC and LN shoot tips of sensitive species like *P. chinense* [55]. This study also indicates that ammonium-induced oxidative stress occurred mainly at the stage of cryoprotection with VSs (LNC) before cooling and warming in LN.

Further optimization of the developed cryopreservation procedure was performed through vitalization of donor plants including using apical sections instead of nodal cuttings for propagation and a liquid medium overlay on top of the solid medium. Shoot tips excised from these donor plants were able to withstand longer osmoprotection treatment (40 min instead of 20 min) and longer exposure to VSs (up to 60 min), which ensured better cryoprotection and resulted in higher (64.2%) regrowth after cryopreservation. The physiological condition of donor plant material usually plays a vital role in the success of cryopreservation. With the endangered plant *Castilleja levisecta*, for example, shoot tips excised from 6- or 12-day-old in vitro plants showed better response to cryopreservation than shoot tips from older plant material [15]. In *Chrysanthemum morifolium* cv. Peak, the optimal age of donor plants for cryopreservation was 4–5.5 weeks for apical shoot tips and 7 weeks for axillary shoot tips [56].

## 5. Conclusions

In this study, we established, for the first time, the procedure for the in vitro seed germination and multiplication of *Penthorum chinense* using node cuttings and developed cryopreservation of the shoot tips as complimentary options for ex situ conservation of this valuable medicinal species. The results highlight that *P. chinense* shoots were very sensitive to osmotic stress and chemical toxicity induced by highly concentrated vitrification solutions. The highest survival and regeneration of both cryoprotected and cryopreserved shoot tips were obtained following 40 min osmoprotection and treatment with alternative vitrification solution B5-85% for 60 min. Regeneration of 64.2% after cryopreservation can be considered relatively high for the wetland species. 

A three-step regrowth starting with ammonium-free medium during the initial five days was critically important for the regeneration of healthy plantlets both from cryoprotected and cryopreserved shoot tips. The results open the door for the more effective conservation of this species and highlight the importance of alternative vitrification solutions, the vigor of donor plants and regrowth on ammonium-free medium for species habituating in the wetlands and thus sensitive to severe osmotic and oxidative stressed provoked by cryopreservation. Considering the complexity of diverse factors during the course of in vitro propagation, cryopreservation and regrowth, machine learning can be applied in modeling, predicting, and optimizing the procedure for a new species [57].

## Figures and Tables

**Table 1:** Experimental design applied in cryopreservation of *Penthorum chinense* shoot tips using a systematic approach in the droplet-vitrification procedure (standard procedure and 15 additional treatments).

Protocol Step	Treatments	Treatment Code
Preculture	No preculture	No-PC
	10% sucrose 31 h → 25% sucrose 17 h	S-10% → S-25%
	10% sucrose 31 h → 17.5% sucrose 17 h	S-10% → S-17.5%, standard
	10% sucrose 48 h	S-10%
Osmoprotection and container	No osmoprotection	No-OP
	C4-35% 20 min, Aluminum foil strips	OP/foil, standard
	C4-35% 20 min, Cryovial (2 mL)	Vial
Cryoprotection (Vitrification solution, VS)	A1-73.7% (PVS2) ice, 30 min	A1-73.7% (PVS2, 30 min)
	A3-90% ice, 30 min	A3-90% (30 min)
	A3-80% ice, 30 min	A3-80% (30 min)
	A3-70% ice, 30 min	A3-70% (30 min), standard
	A3-70% ice, 60 min	A3-70% (60 min)
	B1-100 (PVS3) 25 °C, 30 min	B1-100% (PVS3, 30 min)
	B5-80% 25 °C, 30 min	B5-80% (30 min)
Regrowth	RM1, 5d, dark → RM2, 3w2d, 1 L → MSF,2w, 2 L	RM1-RM2-MSF, standard
	RM2, 5d, dark → RM2, 3w2d, 1 L → MSF, 2w, 2 L	RM2-RM2-MSF
	RM1, 5d, dark → RM2, 1w2d, 1 L → RM2, 1w, 2 L → MSF, 1w, 2 L	RM1-RM2-RM2-MSF
	RM1, 5d, dark → RM2, 3w2d, 1 L → MSF, 2w, 2 L → MSF, 2w, 2 L	RM1-RM2-MSF-MSF
	RM2, 5d, dark → RM2, 3w2d, 1 L → RM2, 2w, 2 L	RM2~

RM1—MS medium without NH_4_NO_3_ + casein hydrolysate 1 g L^−1^ + GA_3_ 1 mg L^−1^ + BA 0.5 mg L^−1^; RM2—MS medium + casein hydrolysate 1 g L^−1^ + GA_3_ 1 mg L^−1^ + BA 0.5 mg L^−1^; MSF—MS medium without growth regulators; RM2~—no medium change; d, days; w, weeks; 1L and 2L, light provided by 1 and 2 fluorescent lamps (40 and 60 µE m^−2^ s^−1^, respectively); “Standard” indicates treatments composing the standard procedure where other stages are the same as in the standard protocol.

**Table 2:** Composition of cryoprotectant solutions used for preculture, osmoprotection, cryoprotection and unloading.

Protocol Step	Solutions	Composition (%, w/v)	Total Concentration (%, w/v)
Preculture and unloading	S-10%	S* 10.0	10.0
	S-35%	S 35.0	35.0
Osmoprotection	C4-35%	G 17.5 + S 17.5	35.0
Cryoprotection (vitrification solution)	A3-90%	G 37.5 + DMSO 15.0 + EG 15.0 + S 22.5	90.0
	A1-73.7% (PVS2)	G 30.0 + DMSO 15.0 + EG 15.0 + S 13.7	73.7
	A3-80%	G 33.3 + DMSO 13.3 + EG 13.3 + S 20.1	80.0
	A3-70%	G 29.2 + DMSO 11.7 + EG 11.7 + S 17.4	70.0
	B1-100% (PVS3)	G 50.0 + S 50.0	100.0
	B5-80%	G 40.0 + S 40.0	80.0
	B5-85%	G 42.5 + S 42.5	85.0

* S, sucrose; G, glycerol; DMSO, dimethyl sulfoxide; EG, ethylene glycol. All solutions were made on the basis of MS medium; pH was adjusted to 5.8 before filter-sterilization.

**Table 3:** Height and dry weight of in vitro grown *Penthorum chinense* plantlets depending on growth medium and culture conditions.

No.	Culture Medium	AC	Illumination	Height (cm)		Dry Weight (g)	
				Shoots	Roots	Shoots	Roots
**1**	**1/2MSF**	**+**	2 L	13.2 ± 4.8 ^b,c^	6.5 ± 3.4 ^b^	0.057 ± 0.020 ^a,b^	0.006 ± 0.006 ^b,c^
**2**	**MSF**	**+**	2 L	11.2 ± 3.2 ^c,d^	4.1 ± 2.5 ^c^	0.051 ± 0.010 ^a,b^	0.005 ± 0.004 ^b,c^
**3**	**MSF + Liquid**	**+**	2 L	16.2 ± 3.7 ^b^	8.3 ± 1.8 ^b^	0.066 ± 0.024 ^a^	0.006 ± 0.005 ^b^
**4**	**MSF**	**−**	2 L	21.6 ± 2.3 ^a^	11.6 ± 1.9 ^a^	0.056 ± 0.012 ^a,b^	0.012 ± 0.005 ^a^
**5**	**MS + BA0.7 + NAA1.0**	**+**	2 L	7.9 ± 1.7 ^d^	1.1 ± 0.4 ^d^	0.036 ± 0.007 ^b^	0.001 ± 0.001 ^c^
**6**	**MSF**	**+**	1 L	11.8 ± 3.1 ^c,d^	5.1 ± 2.7 ^c^	0.042 ± 0.007 ^b^	0.002 ± 0.002 ^b,c^
	*Pr < p*			*p* < 0.0001	*p* < 0.0001	*p* < 0.0052	*p* < 0.001

MSF—standard MS medium with 30 g L^−1^ sucrose and 3.5 g L^−1^ gelrite; 1/2MSF—same medium with half-strength mineral salts; MSF + Liquid—overlay of liquid medium on top of the gelled solid medium added at day 10; MS + BA0.7 + NAA1.0—MS medium with 30 g L^−1^ sucrose, 3.5 g L^−1^ gelrite, 0.7 mg L^−1^ benzyl adenine (BA) and 1.0 mg L^−1^ α-naphthaleneacetic acid (NAA); AC—activated charcoal (1.0 g L^−1^); 1 L and 2 L—illumination of 40 and 60 µE m^−2^ s^−1^, provided by 1 or 2 fluorescent lamps, respectively; Means with the same letters (a,b,c,d) in each column are not significantly different by Least Significant Difference Test (LSDT, *p* < 0.05).

**Table 4:** Effect of preculture treatments on survival and regeneration of cryoprotected control (LNC) and cryopreserved (LN) *Penthorum chinense* shoot tips.

Preculture Treatments	LNC		LN	
	Survival	Regeneration	Survival	Regeneration
No-PC	71.1 ± 9.6 ^a,b^	36.4 ± 15.2 ^a^	73.3 ± 9.6 ^a^	33.6 ± 13.8 ^a^
S-10% → S-25%	35.1 ± 9.4 ^c^	18.7 ± 7.4 ^a^	29.3 ± 8.6 ^b^	8.2 ± 4.1 ^b^
S-10% → S-17.5%, standard	81.8 ± 7.3 ^a^	35.0 ± 8.7 ^a^	67.6 ± 14.4 ^a^	35.0 ± 11.2 ^a^
S-10%	67.3 ± 7.4 ^b^	35.0 ± 5.0 ^a^	61.3 ± 11.3 ^a^	30.0 ± 7.1 ^a^
*Pr < p*	*p <* 0.0001	ns	*p* < 0.0052	*p* < 0.05

No-PC, no preculture; S-10% → S-25%, 10% sucrose (S-10%) and 25% sucrose (S-25%) for 31 h and 17 h, respectively; S-10% → S-17.5%, 10% sucrose (S-10%) and 17.5% sucrose (S-17.5%) for 31 h and 17 h; S-10%, 10% sucrose for 48 h. After preculture, shoot tips were osmoprotected with C4-35% for 20 min, and cryoprotected with VS A3-70% on ice for 30 min before storage in LN; Means with the same letters (a,b,c) in each column are not significantly different by Least Significant Difference Test (*p* < 0.05); ns, non-significant.

**Table 5:** Effect of osmoprotection (OP) and cooling/warming container on survival and regeneration of cryoprotected control (LNC) and cryopreserved (LN) *Penthorum chinense* shoot tips.

Osmoprotection and Container	LNC		LN	
	Survival	Regeneration	Survival	Regeneration
No-OP	52.2 ± 10.5 ^b^	13.3 ± 9.4 ^b^	46.9 ± 9.6 ^a^	16.7 ± 12.5 ^a^
OP/foil, standard	81.8 ± 7.3 ^a^	35.0 ± 8.7 ^a^	67.6 ± 14.4 ^a^	35.0 ± 11.2 ^a^
Vial	-	-	22.4 ± 13.2 ^b^	12.5 ± 13.0 ^a^
*Pr < p*	*p* < 0.05	*p* < 0.05	*p* < 0.05	ns

No-OP, no osmoprotection; OP/foil, standard, osmoprotection with C4-35% for 20 min, and using the aluminum foil strips for cooling and rewarming; Vial, osmoprotection with C4-35% for 20 min, and using the 2 mL cryovial; Means with the same letters (a,b) in each column are not significantly different by Least Significant Difference Test (*p* < 0.05); ns, non-significant.

**Table 6:** Effect of cryoprotection treatments (vitrification solution) on the survival and regeneration of cryoprotected control (LNC) and cryopreserved (LN) *Penthorum chinense* shoot tips.

Cryoprotection Treatments	LNC		LN	
	Survival	Regeneration	Survival	Regeneration
A1-73.7% (PVS2), 30 min	64.2 ± 9.8 ^a^	35.8 ± 15.1 ^a^	68.9 ± 7.3 ^a^	41.1 ± 9.9 ^a^
A3-90%, 30 min	75.8 ± 7.1 ^a,b^	44.2 ± 8.9 ^a^	73.9 ± 5.7 ^a^	39.3 ± 6.4 ^a^
A3-80%, 30 min	70.0 ± 8.2 ^a,b^	44.2 ± 12.7 ^a^	64.6 ± 9.3 ^a^	36.8 ± 11.6 ^a^
A3-70%, 30 min, standard	88.3 ± 11.2 ^a^	48.3 ± 25.2 ^a^	71.2 ± 8.8 ^a^	49.8 ± 14.6 ^a^
A3-70%, 60 min	60.0 ± 11.5 ^b^	31.1 ± 11.1 ^a^	59.0 ± 10.7 ^a^	26.2 ± 6.2 ^a^
B1-100 (PVS3), 30 min	68.9 ± 14.6 ^a,b^	29.2 ± 11.8 ^a^	75.2 ± 7.8 ^a^	32.5 ± 11.8 ^a^
B5-80%, 30 min	78.3 ± 6.9 ^a,b^	45.8 ± 13.8 ^a^	70.2 ± 9.3 ^a^	40.9 ± 12.8 ^a^
*Pr < p*	*p < 0.05*	ns	ns	ns

See Table 2 for vitrification solution composition. Means with the same letters (a,b) in each column are not significantly different by Least Significant Difference Test (*p* < 0.05); ns, non-significant.

**Table 7:** Effect of regrowth treatments on survival and regeneration of cryoprotected control (LNC) and cryopreserved (LN) *Penthorum chinense* shoot tips.

Regrowth Medium	LNC		LN	
	Survival	Regeneration	Survival	Regeneration
RM1-RM2-MSF, standard	100.0 ± 0.0 ^a^	43.6 ± 8.8 ^a^	73.9 ± 2.6 ^a^	35.0 ± 4.8 ^a^
RM2-RM2-MSF	70.7 ± 0.4 ^b^	10.0 ± 5.8 ^b^	56.9 ± 5.7 ^b^	5.2 ± 3.4 ^b^
RM1-RM2-RM2-MSF	96.7 ± 3.3 ^a^	49.0 ± 5.0 ^a^	78.8 ± 0.7 ^a^	35.6 ± 5.1 ^a^
RM1-RM2-MSF-MSF	97.5 ± 2.5 ^a^	45.0 ± 4.1 ^a^	83.9 ± 3.7 ^a^	39.4 ± 3.6 ^a^
RM2~ (no medium change)	70.7 ± 0.4 ^b^	0.0 ± 0.0 ^b^	57.8 ± 7.2 ^b^	3.3 ± 3.3 ^b^
*Pr < p*	*p < 0.05*	*p < 0.05*	*p < 0.05*	*p < 0.05*

RM1—MS medium without NH_4_NO_3_ + casein hydrolysate 1 g L^−1^ + GA_3_ 1 mg L^−1^ + BA 0.5 mg L^−1^ + AC 1 g L^−1^; RM2—MS medium + casein hydrolysate 1 g L^−1^ + GA_3_ 1 mg L^−1^ + BA 0.5 mg L^−1^; MSF—MS medium without growth regulators. Means with the same letters (a,b) are not significantly different by Least Significant Difference Test (*p* < 0.05).

## Data Availability

Not applicable.

## References

[1] The Plant List: A Working List of All Plant Species. http://www.theplantlist.org/.

[2] Mahesh T., Menon V.P. (2004). Quercetin alleviates oxidative stress in streptozotocin-induced diabetic rats. Phytother. Res..

[3] Moon Y.J., Wang X., Morris M.E. (2006). Dietary flavonoids: Effects on xenobiotic and carcinogen metabolism. Toxicol. Vitro.

[4] Kang D., Lundström A., Steiner H. (1996). Trichoplusia ni attacin A, a differentially displayed insect gene coding for an antibacterial protein. Gene.

[5] Sigurdsson S., Nordmark G., Göring H.H., Lindroos K., Wiman A.C., Sturfelt G., Jönsen A., Rantapää-Dahlqvist S., Möller B., Kere J. (2005). Polymorphisms in the tyrosine kinase 2 and interferon regulatory factor 5 genes are associated with systemic lupus erythematosus. Am. J. Hum. Genet..

[6] (1993). Dictionary of Traditional Chinese Materia Medica (Chinese-English).

[7] Shin H.J. (2011). Ecological study of habitat restoration and environmental characteristics of *Pethorum. chinense*. Ph.D. Thesis.

[8] Northcutt C., Davies D., Gagliardo R., Bucalo K., Determann R.O., Cruse-Sanders J.M., Pullman G.S. (2012). Germination in vitro, micropropagation, and cryogenic storage for three rare pitcher plants: *Sarracenia. oreophila* (Kearney) Wherry (federally endangered), *S. leucophylla* Raf., and *S. purpurea* spp. *venosa* (Raf.) Wherry. HortScience.

[9] Pence V.C., Ballesteros D., Walters C., Reed B.M., Philpott M., Dixon K.W., Pritchard H.W., Culley T.M., Vanhove A.C. (2020). Cryobiotechnologies: Tools for expanding long-term ex situ conservation to all plant species. Biol. Conserv..

[10] Kaviani B., Kulus D. (2022). Cryopreservation of endangered ornamental plants and fruit crops from tropical and subtropical regions. Biology.

[11] Tanaka D., Niino T., Uemura M. (2018). Cryopreservation of plant genetic Resources. Adv. Exp. Med. Biol..

[12] Kim H.H., Lee S.C. (2012). Personalisation of droplet-vitrification protocols for plant cells: A systematic approach to optimising chemical and osmotic effects. CryoLetters.

[13] Shin D.J., Kong H., Popova E.V., Moon H.K., Park S.Y., Park S.U., Lee S.C., Kim H.H. (2012). Cryopreservation of *Kalopanax. septemlobus* embryogenic callus using vitrification and droplet-vitrification. CryoLetters.

[14] Rathwell R., Popova E., Shukla M.R., Saxena P.K. (2016). Development of cryopreservation methods for cherry birch (*Betula. lenta* L.), an endangered tree species in Canada. Can. J. For. Res..

[15] Salama A., Popova E., Jones M.P., Shukla M.R., Fisk N.S., Saxena P.K. (2018). Cryopreservation of the critically endangered golden paintbrush (*Castilleja. levisecta* Greenm.): From nature to cryobank to nature. In Vitro Cell. Dev. Biol.-Plant.

[16] Choi C.H., Popova E., Lee H., Park S.U., Ku J., Kang J.H., Kim H.H. (2019). Cryopreservation of endangered wild species, *Aster. altaicus* var. uchiyamae Kitam, using droplet-vitrification procedure. CryoLetters.

[17] Popova E.V., Shukla M.R., McIntosh T., Saxena P.K. (2021). In vitro and cryobiotechnology approaches to safeguard *Lupinus rivularis* Douglas ex Lindl., an endangered plant in Canada. Agronomy.

[18] Turner S.R., Touchell D.H., Senaratna T., Bunn E., Tan B., Dixon K.W. (2001). Effects of plant growth regulators on survival and recovery growth following cryopreservation. CryoLetters.

[19] Bettoni J.C., Kretzschmar A.A., Bonnart R., Shepherd A., Volk G.M. (2019). Cryopreservation of 12 *Vitis* species using apical shoot tips derived from plants grown in vitro. HortScience.

[20] Murashige T., Skoog F. (1962). A revised medium for rapid growth and bio assays with tobacco tissue cultures. Physiol Plant.

[21] Pullman G.S., Skryabina A. (2007). Liquid medium and liquid overlays improve embryogenic tissue initiation in conifers. Plant Cell Rep..

[22] Hussien F.A., Osman M.A., Idris T.I. (2014). The influence of liquid media support, gelling agents and liquid overlays on performance of in vitro cultures of ginger (*Zingiber. officinale*). Intl. J. Sci. Res. Pub..

[23] Jain A., Poling M.D., Smith A.P., Nagarajan V.K., Lahner B., Meagher R.B., Raghothama K.G. (2009). Variations in the composition of gelling agents affect morphophysiological and molecular responses to deficiencies of phosphate and other nutrients. Plant Physiol..

[24] Das N., Tripathi N., Basu S., Bose C., Maitra S., Khurana S. (2015). Progress in the development of gelling agents for improved culturability of microorganisms. Front. Microbiol..

[25] Thomas T.D. (2008). The role of activated charcoal in plant tissue culture. Biotechnol. Adv..

[26] Pacek-Bieniek A., Dyduch-Siemińska M., Rudaś M. (2010). Influence of activated charcoal on seed germination and seedling development by asymbiotic method in *Zygostates. grandiflora* (Lindl.) Mansf. (Orchidaceae). Folia Hortic..

[27] Polzin F., Sylvestre I., Déchamp E., Ilbert P., Etienne H., Engelmann F. (2014). Effect of activated charcoal on multiplication of African yam (*Dioscorea. cayenensis.-rotundata*) nodal segments using a temporary immersion bioreactor (RITA®). In Vitro Cell. Dev. Biol.-Plant.

[28] Kim J.K., Park S.U. (2017). Enhancement of in vitro rooting through growth media, gelling agents and activated charcoal in *Lycium. Chinense*. Online J. Biol. Sci..

[29] Shin S.L., Lee C.H. (2011). Effect of medium components and culture methods on shoot regeneration from *Athyrium niponicum*. Kor. J. Plant Res..

[30] Kuriyama A., Kuriyama K., Kuriyama F., Kuriyama M. (1996). Sensitivity of cryopreserved *Lavandula vera* cells to ammonium ion. J. Plant Physiol..

[31] Pullman G.S., Gupta P.K., Timmis R., Carpenter C., Kreitinger M., Welty E. (2005). Improved Norway spruce somatic embryo development through the use of abscisic acid combined with activated carbon. Plant Cell Rep..

[32] Sakai A., Engelmann F. (2007). Vitrification, encapsulation-vitrification and droplet-vitrification: A review. CryoLetters.

[33] Carpentier S.C., Witters E., Laukens K., van Onckelen H., Swennen R., Panis B. (2007). Banana (*Musa* spp.) as a model to study the meristem proteome: Acclimation to osmotic stress. Proteomics.

[34] Hitmi A., Barthomeuf C., Sallanon H. (1999). Cryopreservation of *Chrysanthemum cinerariaefolium* shoot tips. Effects of pretreatment conditions and retention of biosynthetic capacity. CryoLetters.

[35] Suzuki M., Ishikawa M., Okuda H., Noda K., Kishimoto T., Nakamura T., Ogiwara I., Shimura I., Akihama T. (2006). Physiological changes in gentian axillary buds during two-step preculturing with sucrose that conferred high levels of tolerance to desiccation and cryopreservation. Ann. Bot..

[36] Zhu G.Y., Geuns J.M., Dussert S., Swennen R., Panis B. (2006). Change in sugar, sterol and fatty acid composition in banana meristems caused by sucrose-induced acclimation and its effects on cryopreservation. Physiol Plant.

[37] Jitsuyama Y., Suzuki T., Harada T., Fujikawa S. (2002). Sucrose incubation increases freezing tolerance of asparagus (*Asparagus officinalis* L.) embryogenic cell suspensions. CryoLetters.

[38] Lee H.E., Popova E., Park H.N., Park S.U., Kim H.H. (2021). Optimization of a cryopreservation method for the endangered Korean species *Pogostemon yatabeanus* using a systematic approach: The key role of ammonium and growth regulators. Plants.

[39] Mallón R., Bunn E., Turner S.R., González M.L. (2008). Cryopreservation of *Centaurea. ultreiae* (Compositae) a critically endangered species from Galicia (Spain). CryoLetters.

[40] Rall W.F., Fahy G.M. (1985). Ice-free cryopreservation of mouse embryos at −196 °C by vitrification. Nature.

[41] Benson E.E., Reed B.M., Brennan R.M., Clacher K.A., Ross D.A. (1996). Use of thermal analysis in the evaluation of cryopreservation protocols for *Ribes nigrum* L. germplasm. CryoLetters.

[42] Volk G.M., Maness N., Rotindo K. (2004). Cryopreservation of garlic (*Allium sativum* L.) using plant vitrification solution 2. CryoLetters.

[43] Matsumoto T., Sakai A., Takahashi C., Yamada K. (1995). Cryopreservation of in vitro-grown apical meristems of wasabi (*Wasabia japonica*) by encapsulation-vitrification method. CryoLetters.

[44] Kim H.H., Lee Y.G., Park S.U., Lee S.C., Baek H.J., Cho E.G., Engelmann F. (2009). Development of alternative loading solutions in droplet-vitrification procedures. CryoLetters.

[45] Lambert E., Goossens A., Panis B., van Labeke M.C., Geelen D. (2009). Cryopreservation of hairy root cultures of *Maesa lanceolata* and *Medicago truncatula*. Plant Cell Tissue Organ Cult..

[46] Park S.U., Kong H., Shin D.J., Bae C.H., Lee S.C., Bae C.H., Rha E.S., Kim H.H. (2014). Development of vitrification protocol in *Rubia akane* (Nakai) hairy roots using a systematic approach. CryoLetters.

[47] Fahy G.M., MacFarlane D.R., Angell C.A., Meryman H.T. (1984). Vitrification as an approach to cryopreservation. Cryobiology.

[48] Sakai A., Kobayashi S., Oiyama I. (1990). Cryopreservation of nucellar cells of navel orange (*Citrus. sinensis.* Osb. var. *brasiliensis.* Tanaka) by vitrification. Plant Cell Rep..

[49] Kim H.H., Lee Y.G., Shin D.J., Ko H.C., Gwag J.G., Cho E.G., Engelmann F. (2009). Development of alternative plant vitrification solutions in droplet-vitrification procedures. CryoLetters.

[50] Kim H.H., Popova E.V., Shin D.J., Bae C.H., Baek H.J., Park S.U., Engelmann F. (2012). Development of a droplet-vitrification protocol for cryopreservation of *Rubia. akane* (Nakai) hairy roots using a systematic approach. CryoLetters.

[51] Kim H.H., Lee J.K., Yoon J.W., Ji J.J., Nam S.S., Hwang H.S., Cho E.G., Engelmann F. (2006). Cryopreservation of garlic bulbil primordia by the droplet-vitrification procedure. CryoLetters.

[52] Touchell D., Turner S.R., Senaratna T., Bunn E., Dixon K.W. (2002). Cryopreservation of Australian Species—The Role of Plant Growth Regulators. Cryopreservation of Plant Germplasm II Biotechnology in Agriculture and Forestry.

[53] Mukherjee P., Mandal B.B., Bhat K.V., Biswas A.K. (2009). Cryopreservation of Asian *Dioscorea. bulbifera* L. and *D. alata* L. by vitrification: Importance of plant growth regulators. CryoLetters.

[54] Kuriyama A., Watanabe K., Ueno S., Mitsuda H. (1989). Inhibitory effect of ammonium ion on recovery of cryopreserved rice cells. Plant Sci..

[55] Pennycooke J.C., Towill L.E. (2001). Medium alterations improve regrowth of sweet potato (*Ipomoea. batatas* [L.] lam.) shoot tips cryopreserved by vitrification and encapsulation-dehydration. CryoLetters.

[56] Lee Y.G., Popova E., Cui H.Y., Kim H.H., Park S.U., Bae C.H., Lee S.C., Engelman F. (2011). Improved cryopreservation of chrysanthemum (*Chrysanthemum. morifolium*) using droplet-vitrification. CryoLetters.

[57] Hesami M., Naderi R., Tohidfar M. (2020). Introducing a hybrid artificial intelligence method for high-throughput modeling and optimizing plant tissue culture processes: The establishment of a new embryogenesis medium for chrysanthemum, as a case study. Appl. Microbiol. Biotechnol..

